# 

**Published:** 1861-04

**Authors:** 


### THE MEDICAL CRITIC AND PSYCHOLOGICAL JOURNAL.

EDITED BY

FORBES WINSLOW, M.D., D.C.L., Oxon.

**No. II.—APRIL, 1861.**

TO BE CONTINUED QUARTERLY.

LONDON:
JOHN W. DAVIES, 54, PRINCE'S STREET,
LEICESTER SQUARE.
EDINBURGH: MACLACHLAN AND CO. DUBLIN: FANNIN AND CO.

*Price Three Shillings and Sixpence.*

